# Automated quantification of fluorescence and morphological changes in pretreated wood cells by fluorescence macroscopy

**DOI:** 10.1186/s13007-023-00991-6

**Published:** 2023-02-15

**Authors:** Edwige Audibert, Berangère Lebas, Corentin Spriet, Anouck Habrant, Brigitte Chabbert, Gabriel Paës

**Affiliations:** 1 Université de Reims Champagne-Ardenne, INRAE, FARE, UMR A 614, Reims, France; 2 Univ. Lille, CNRS, UMR 8576 – UGSF - Unité de Glycobiologie Structurale Et Fonctionnelle, Lille, France; 3 Univ. Lille, CNRS, Inserm, CHU Lille, Institut Pasteur de Lille, US 41 - UMS 2014 - PLBS, Lille, France

**Keywords:** Morphology, Fluorescence, Cell, Plant, Quantification, Macroscopy, Automation

## Abstract

**Background:**

Lignocellulosic biomass is a complex network of polysaccharides and lignin that requires a pretreatment step to overcome recalcitrance and optimize valorisation into biobased products. Pretreatment of biomass induces chemical and morphological changes. Quantification of these changes is critical to understand biomass recalcitrance and to predict lignocellulose reactivity. In this study, we propose an automated method for the quantification of chemical and morphological parameters through fluorescence macroscopy, which was applied on wood samples (spruce, beechwood) pretreated with steam explosion.

**Results:**

Results in fluorescence macroscopy highlighted the impact of steam explosion on spruce and beechwood: fluorescence intensity of samples was highly altered, especially for the most severe conditions. Morphological changes were also revealed: shrinkage of cells and deformation of cell walls manifested as the loss of rectangularity or circular shape, for tracheids in spruce and vessels in beechwood respectively. Quantification of fluorescence intensity of cell walls and quantification of morphological parameters related to cell lumens were carried out accurately by applying the automated method onto the macroscopic images. The results showed that lumens area and circularity could be considered as complementary markers of cell deformation, and that fluorescence intensity of the cell walls could be related to morphological changes and to the conditions of pretreatment.

**Conclusions:**

The developed procedure allows simultaneous and effective quantification of morphological parameters and fluorescence intensity of the cell walls. This approach can be applied to fluorescence macroscopy as well as other imaging techniques and provides encouraging results towards the understanding of biomass architecture.

**Supplementary Information:**

The online version contains supplementary material available at 10.1186/s13007-023-00991-6.

## Background

Lignocellulosic biomass, such as wood, dedicated crops and agricultural residues, is a renewable feedstock with a high potential to develop new processes of biobased products for chemistry, energy and materials [1]. Indeed, lignocellulosic biomass is mainly composed of polysaccharides (cellulose and hemicelluloses) and polyphenols (lignin). Together they form a complex network making the plant cell wall structure [2], which can be observed by several imaging techniques. Analyses and quantification of parameters from cell walls imaging are essential when studying lignocellulosic biomass and require the use of appropriate methods.

When transforming lignocellulosic biomass in biorefinery processes, recalcitrance of plant cell walls to conversion into bioproducts must be overcome, that is why a pretreatment step is required [3] and it is considered as a critical step. Several pretreatment technologies have been developed and can be categorized into biological, physical, chemical and physico-chemical methods [4]. Steam explosion enters the last category of pretreatments and is known as an efficient, environmentally friendly and cost-effective technology [5]. During steam explosion pretreatment, lignocellulosic biomass is first exposed to high-pressure saturated steam then the pressure is promptly reduced to atmospheric pressure, resulting in the explosion of the materials [6, 7]. This leads to decomposition of hemicelluloses and redistribution of lignin in the lignocellulosic network, alongside with cell morphological changes [8, 9].

Among all the markers of lignocellulosic biomass recalcitrance that have been investigated, none are universal [10–14] and few are related to cell morphology changes after physical or biological treatments [15]. However, relating morphological markers to pretreatment intensity is critical to better understand its mechanism and to predict the reactivity of lignocellulose. Previously, some research groups have quantified some morphological markers at the cellular scale such as: the cell circularity and the cell diameter in wood, based on X-ray tomography [16]; the cell lumen area and wall thickness in maize internodes using optical microscopy [17]; the granulometric size distribution to study cellular morphology in maize internodes based on macroscopic images [18]. Even if relevant, these methods are not fully automated and are limited to a few types of samples.

Another measurement of interest when studying pretreatment of lignocellulosic biomass is the fluorescence of cell walls. It has been proved that hydrothermal pretreatments have an impact on fluorescence intensity which can be quantified either by spectrofluorimetry [19] or confocal microscopy [11]. However, these methods are quite time-consuming even if accurate.

Automation is a key condition and is already used to process images in microscopy. Indeed, automated procedures have been developed for histological recognition [20], wood recognition [21] or to follow lignification of plant cell walls [22] but not to quantify morphological and fluorescence parameters.

Here, we have developed an automated method which is quick to handle in order to quantify, for instance, fluorescence and structural changes following pretreatment of wood, based on images acquired by fluorescence macroscopy. Advantages of the developed approach are easiness of sample preparation and short time steps associated to sample preparation, image acquisition and data analysis. Moreover, compared to fluorescence confocal microscopy, the broader field of view of the fluorescence macroscope gives a more representative view of the samples and high resolution allows to appreciate small details of the samples. The method proposed here has been validated on two different wood species of ecological, technical and economical interest in Europe and France: spruce and beechwood, which are widespread across Europe [23, 24] and count for 8% and 10% of French forestry, respectively [25]. In order to generate contrasted samples, they have been pretreated by steam explosion at different levels of severity.

## Methods

### Wood sample preparation

Spruce (*Picea abies*) and beechwood (*Fagus sylvatica*) were collected from forests of the Région Grand Est in France. Spruce and beechwood were cut into cubes with edges of 2 cm. These samples were kept at room temperature for 72 h to reach equilibrium humidity. Then they were pretreated in a batch mode using steam explosion at LERMAB (Nancy, France) [26]. Samples were pre-soaked in distilled water using vacuum-pressure with vacuum break every hour during 7 h and 2 h for spruce and beechwood, respectively. This step was followed with one night of soaking in distilled water at atmospheric pressure. Steam explosion was carried out at 3 different severity conditions by varying temperature (170 °C, 190 °C and 210 °C) and at a constant residence time of 15 min. Five samples as replicates were pretreated for each condition. To remove soluble fraction, pretreated samples were washed twice in 150 mL of deionised water for 1 h, and then dried for 72 h at 40 °C. Drying step was mandatory to prepare sample for microscopy analyses and avoid the use of resins that can have an impact on the material [27]. The dried samples were cut into fragments of 0.5 cm width, 2 cm long and 0.5 cm thickness using razor blades. Transverse sections of 30 µm thickness of untreated (raw) and pretreated samples were finally prepared using a microtome equipped with disposable blades. Sections were mounted in deionised water between a cover glass and a cover-slip.

### Fluorescence macroscopy

Images were acquired at a 63 × total magnification using the Axio Zoom V16 fluorescence macroscope (Zeiss, Germany) equipped with an 8-bit RGB camera with a numerical aperture of 0.25. For each acquisition, identical settings were used. Field of view, resolution and depth of field were automatically calculated and were equal to 3.7 mm, 0.8 µm and 12 µm, respectively. Excitation of the sections was performed at 353 nm with an exposure time varying between 100 and 3000 ms (100 ms step) to acquire images from underexposure to overexposure. Multi-exposure was used here since fluorescence intensities were dependent of pretreatment severity and were different for each sample at the same exposure time. A 420–470 nm bandpass filter was used for the emission.

For each pretreatment condition as well as for the untreated samples, images were acquired on five different areas of five different samples, except for spruce samples pretreated at 210 °C as only one section was available due to the severity of pretreatment damaging the samples. Moreover, acquisition of images was repeated on several samples and on different days to take into account possible technical variability. Images acquired had a size of 4096 × 3008 pixels and allowed to observe thousands of tracheids for spruce and hundreds of vessels for beechwood.

### Image analysis and quantification of fluorescence intensity and morphological parameters

**Fig. 1:**
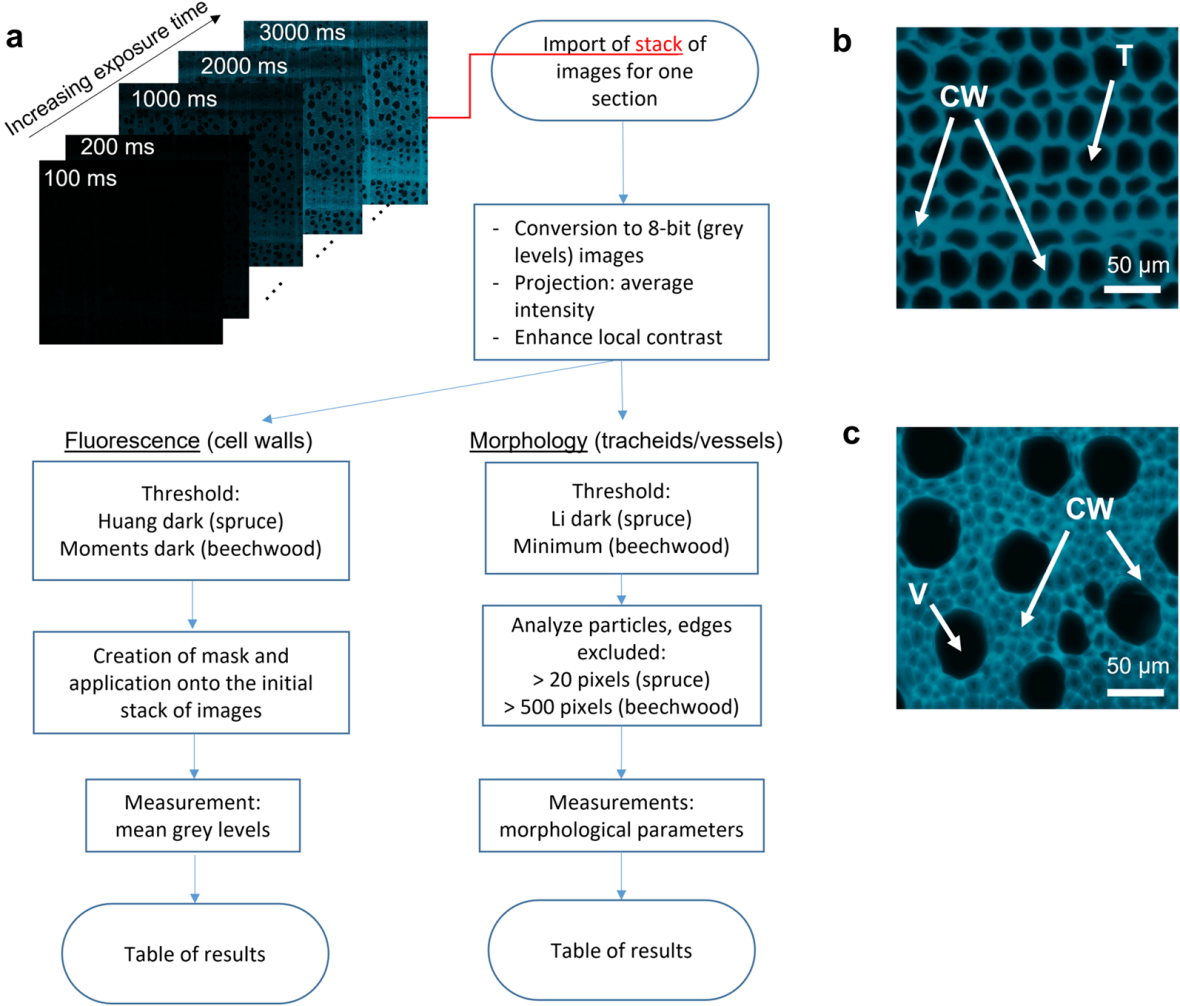
**a** Logical flowchart for measurement of morphological parameters and fluorescence intensity based on fluorescence macroscopy images. **b** Image of spruce cell wall (CW) and tracheids (T). **c** Image of beechwood cell wall (CW) and vessels (V)

The images of five sections per condition were analysed with ImageJ software (version 1.53c). An automated procedure was developed to quantify fluorescence intensity and morphological parameters (Fig. 1). In few seconds, this procedure enables to quantify several parameters inherent to cells. Two procedures have been developed to fit tissular specificities of analysed wood samples and their relevancy: tracheid lumens for spruce and vessel lumens for beechwood.

**Fig. 2:**
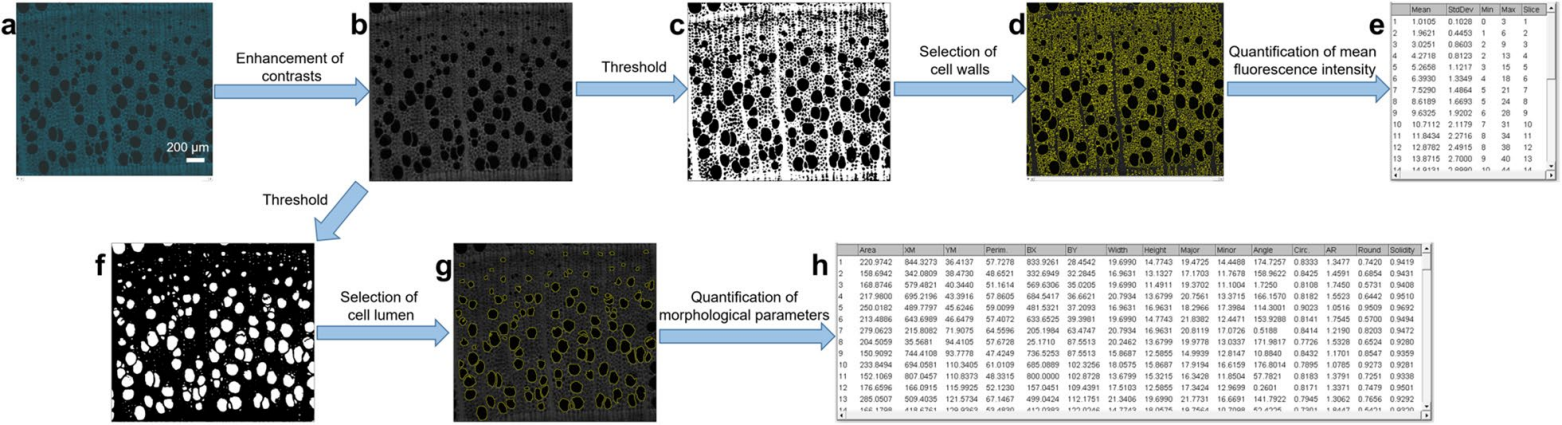
Application of the automated procedure on a beechwood sample. **a** Stack of original images. **b** Enhanced grey level average image. **c** Threshold for fluorescence intensity quantification. **d** Application to the initial stack. **e** Table of results for fluorescence intensity. **f** Threshold for morphological parameters quantification. **g** Selection of the vessels. **h** Table of results for morphological parameters

The analysis procedure is designed to be applied to stacks of images, created from a collection of the same section at different exposure time (Fig. 2a). The latter will be duplicated in 2, allowing on the first one to perform different morphological operations in order to detect different parts of the wood sections without altering the fluorescence. It is then possible to generate morphological masks that can be applied to the initial image stack and thus optimize the detection accuracy while preserving the signal. The whole procedure is performed automatically thanks to a macro developed under ImageJ and whose key steps are the following.

The image, which must be, or be converted in 8 bits gray level (Fig. 2b), is processed by a local contrast enhancement algorithm (CLAHE) in order to compensate the contrast inhomogeneity (due to wavy sections) (Fig. 2c). An automatic threshold, “Huang dark” or “Moments dark” for spruce and beechwood respectively, was performed on an average intensity projection of this image stack, which allows to detect the plant cell walls and to extract a selection (Fig. 2d). This selection is then applied to all the images of the unmodified image stack in order to measure the average intensity of the latter along the image stack (Fig. 2e).

A different threshold, “Li dark” for spruce or “Minimum” for beechwood, was applied on the lowest intensities of the contrasted image (Fig. 2f) and the plugin “Analyze particles” available in ImageJ software was used to select the cells of interest: tracheids or vessels (Fig. 2g). A lower bound for the size of the particles was set (20 pixels for spruce and 500 pixels for beechwood) so that only cells of interest would be analysed. Several morphological parameters inherent to these cells were calculated (Fig. 2h), including area, perimeter and circularity. Area corresponds to the area of the lumen, perimeter is the outline of the lumen, and circularity quantifies if a cell is perfectly rectangular (tracheids of spruce) or round (vessels of beechwood).

### Statistical analysis

Statistical analyses of morphology and fluorescence data were performed using Holm-Sidak test on SigmaPlot (version 12.0).

## Results and discussion

### Fluorescence macroscopy

**Fig. 3:**
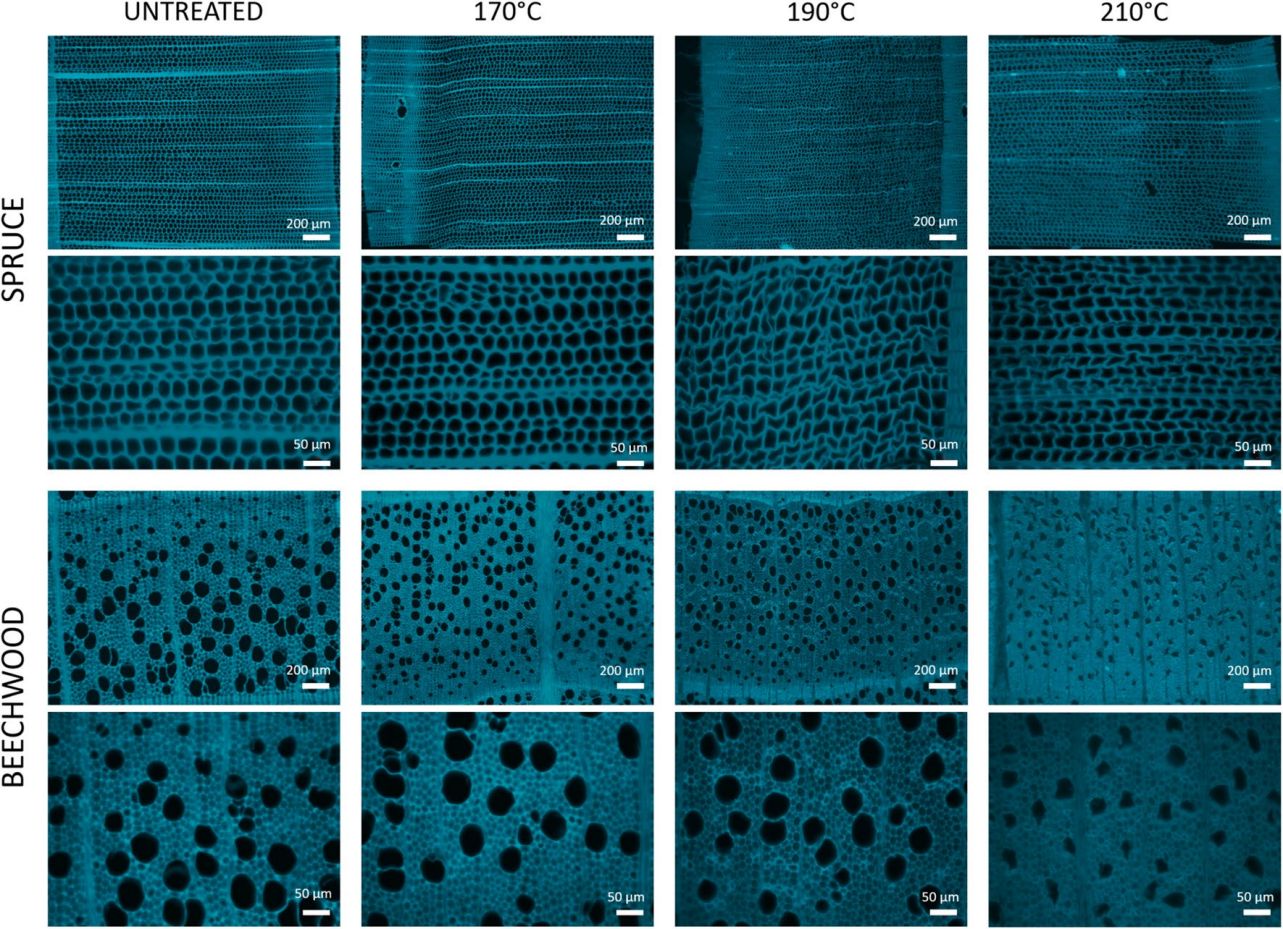
Fluorescence macroscope images of untreated and pretreated (170 °C, 190 °C, 210 °C) spruce and beechwood transverse sections. Brightness was optimised for each image to facilitate comparison due to differences in fluorescence intensity of samples. Optical magnifications 63 × and 258x

Based on past experiments in the literature, the typical ranges of temperature and time for steam explosion pretreatment are 160–260 °C and from several seconds to a few minutes, respectively [28]. Thus, samples of spruce and beechwood were pretreated with steam explosion at three different temperatures (170 °C, 190 °C and 210 °C) for 15 min and the structures of cell walls were studied by imaging transverse sections using fluorescence macroscopy (Fig. 3). As stated earlier, only one section was obtained from the spruce samples pretreated at 210 °C. This unique sample may not be representative as a study from Qian et al. [29] reported that spruce samples were completely shattered under most severe pretreatment conditions. Therefore, in the rest of the manuscript, images acquired for this sample will be used as an example to demonstrate the applicable range of the developed procedure, since quantifications were still possible despite the strong structural modifications. In accordance, the data collected from this extreme sample are not included in any statistical analysis.

Wood autofluorescence originates from the phenolic functions of lignin contained in cell walls which have a broad range of fluorescence emission with a maximum of absorption in UV light [30] so they can be excited at 353 nm. Imaging cell wall autofluorescence offers a suitable view of the structural architecture of cells, without a bias due to the thickness of the sections that can occur when imaging with brightfield light. Moreover, the low magnification offered with macroscopy coupled with high resolution enables to have an overall picture of anatomical changes occurring in the samples.

First, visual observations showed that tracheid cell walls in spruce were deformed for the most severe pretreatment conditions (190 °C and 210 °C), while there seemed to be no visible morphological differences between untreated samples and samples pretreated at 170 °C. For beechwood, a cell shape deformation was noticeable only for 210 °C pretreatment condition. Shrinkage of cells manifested for spruce as less rectangular tracheids and for beechwood as the loss of the circular shape of the vessels. Visual observations also indicate that fluorescence intensity was influenced by the pretreatment and was decreasing when pretreatment intensity was more severe.

### Quantification of morphological parameters

Based on the visual observation of fluorescence macroscopy images, morphological parameters were defined and quantified. The developed procedure focuses on the quantification of perimeter, area and circularity of spruce tracheid lumens and beechwood vessel lumens, as they were found to be deformed with high severity pretreatments. Using images at a 63 × magnification to get a representative selection of cells, the automated procedure was applied to quantify these parameters on thousands of tracheids and hundreds of vessels so that representativeness was achieved.

**Fig. 4:**
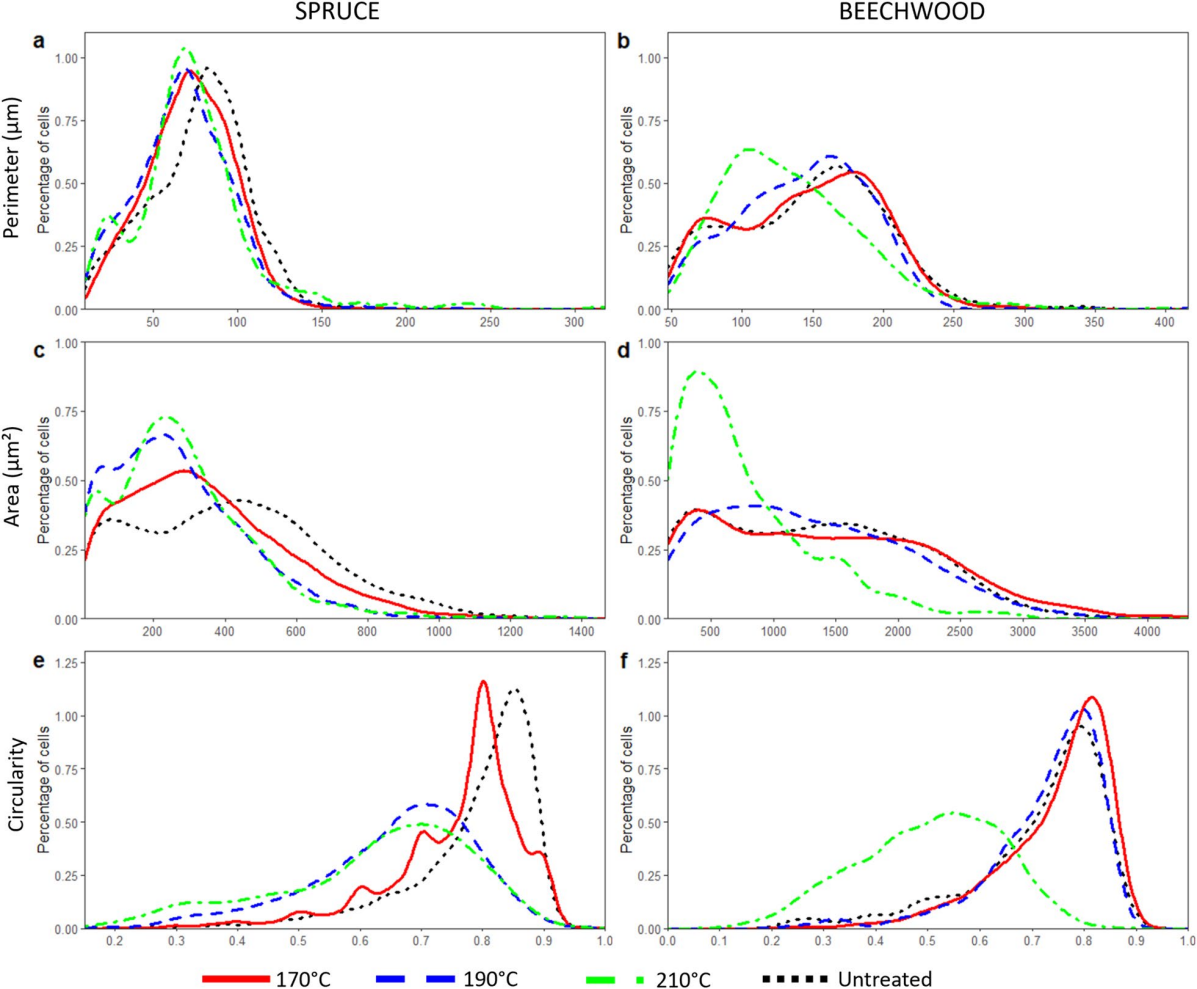
Distribution curves of morphological parameters for each wood species. Distribution of tracheid lumens of spruce according to measurement of perimeter, area and circularity (**a**, **c**, **e**, respectively). Distribution of vessel lumens of beechwood according to measurement of perimeter, area and circularity (**b**, **d**, **f**, respectively)

To evaluate finely the different parameters, distribution of their values was first analysed (Fig. 4).

Mean values and standard deviations of the parameters were also calculated (Table 1). For each pretreatment of each species, the percentages of variation of the data were similar (Additional file 1).

**Table 1:** Mean values and standard deviations of perimeter, area and circularity of cell lumens

Sample	Perimeter (µm)	Area (µm^2^)	Circularity
Spruce—untreated	76 ± 28	414 ± 260	0.79 ± 0.11
Spruce—170 °C	72 ± 26	347 ± 224	0.76 ± 0.11
Spruce—190 °C	68 ± 29	270 ± 190	0.66 ± 0.13
Spruce—210 °C*	73 ± 34	280 ± 201	0.62 ± 0.16
Beechwood—untreated	146 ± 53	1309 ± 796	0.71 ± 0.13
Beechwood—170 °C	145 ± 50	1396 ± 889	0.74 ± 0.11
Beechwood—190 °C	143 ± 46	1292 ± 750	0.73 ± 0.11
Beechwood—210 °C	134 ± 48	776 ± 546	0.50 ± 0.13

^*^one section was analysed instead of 5 for other conditions

The first parameter calculated by image processing was the perimeter of lumens. The distributions were highly similar for spruce samples (Fig. 4a). Based on mean values, no significant differences were found between untreated and pretreated samples of spruce (*p* > *0.05*). For beechwood, only the distribution for the strongest pretreatment was altered (Fig. 4b). Mean of perimeters showed a decrease of 8%, from 146 ± 53 µm (untreated) to 134 ± 48 µm (after treatment at 210 °C), but this was not significant (*p* > *0.05*). Given the lack of significance for mean values, perimeter appears as an unsuitable parameter to quantify structural changes of cell walls, especially in spruce samples. However, when studying the proportion of lumens with a perimeter below 150 µm in beechwood samples, there is a significant increase (*p* < *0.001*) from 49.3% of cells for raw beechwood to 66.9% of cells after pretreatment at 210 °C. Therefore, perimeter of lumens can be considered as a marker of cell shrinkage for beechwood samples pretreated with a high severity.

The second investigated parameter was the area of lumens. Distribution of spruce tracheid lumens (Fig. 4c) revealed there was a higher proportion of lumens below 400 µm^2^ for pretreated samples. The most important change was observed for the samples pretreated at 190 °C for which the area dropped from 414 ± 260 µm^2^ (untreated samples) to 270 ± 190 µm^2^ (*p* = *0.008*). Mean area was reduced from 35% of its initial value. As for beechwood, the distributions of areas of vessel lumens (Fig. 4d) in untreated samples and samples pretreated at 170 °C and 190 °C were almost identical. The important change in the area of vessel lumens could be observed for pretreatment at 210 °C, which was confirmed with mean value of area dropping from 1309 ± 796 µm^2^ for untreated beechwood to 776 ± 546 µm^2^ for 210 °C pretreatment (*p* = *0.007*), corresponding to a 40% decrease. Significant differences were also found between pretreatments at 170 °C and 210 °C (*p* < *0.001*) and between pretreatments at 190 °C and 210 °C (*p* = *0.004*). In regard to these results, area of cell lumens seems to be a marker of morphological changes.

The third parameter of interest was the circularity of lumens, for which the distributions were the most greatly contrasted (Fig. 4e, f). Spruce samples pretreated at 190 °C and 210 °C and beechwood samples pretreated at 210 °C showed a broader distribution than other samples, which implies a decrease in circularity values, demonstrating deformations of cell walls. Loss of circularity was found to be significant for both wood species. For spruce samples, circularity decreased from 0.79 ± 0.11 (untreated) to 0.66 ± 0.13 after pretreatment at 190 °C (*p* < *0.001*), *i.e.* a loss of 16%. For beechwood samples, circularity dropped by almost a third from 0.71 ± 0.13 before pretreatment to 0.50 ± 0.13 after pretreatment at 210 °C (*p* < *0.001*). In comparison to previous results, circularity is likely the best indicator of cell deformation as it is the most statistically significant.

### Quantification of fluorescence intensity

**Fig. 5:**
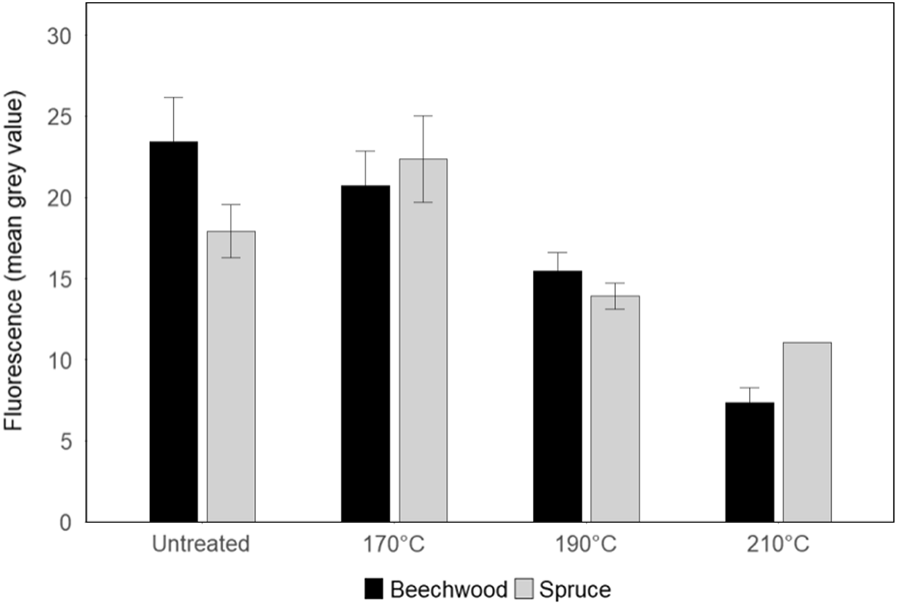
Cell wall fluorescence intensity of untreated and pretreated spruce and beechwood. Values were measured from fluorescence macroscopy images at an exposure time of 2000 ms. See *p*-values in the text for significance of results

Using another capability of the same developed automated procedure, the fluorescence intensity of the samples was quantified from the macroscopy images at a 63 × magnification. It was calculated using grey levels and by computing the mean of grey values of each pixel corresponding to the cell walls on the images, where the mean of grey values corresponds to average fluorescence intensity. The evolution of fluorescence intensity for the untreated and pretreated samples of spruce and beechwood at a constant exposure time of 2000 ms is depicted in Fig. 5.

Loss of fluorescence was noted for the majority of pretreated samples. For spruce samples, fluorescence intensity slightly increased for sample pretreated at 170 °C but not significantly (*p* = *0.178*). However, a significant decrease could be seen between pretreatments at 170 °C and 190 °C (*p* < *0.001*). For beechwood samples, there was a gradual decrease in fluorescence intensity with the temperature of pretreatment. It dropped significantly from 23.44 ± 2.74 for untreated samples to 15.49 ± 1.13 after treatment at 190 °C (*p* = *0.01*), and to 7.34 ± 0.94 after treatment at 210 °C (< *0.001*). The most important decrease was for samples pretreated at 210 °C as intensity was also significantly lower than other pretreatments conditions (*p* < *0.001* after pretreatment at 170 °C and *p* = *0.007* after pretreatment at 190 °C). Results were consistent with visual observations but allowed a more precise quantification of these changes. Furthermore, strong negative correlation between fluorescence intensity and temperature of pretreatment was calculated: r = −0.99 for beechwood, emphasizing the impact of steam explosion on the chemical composition of lignocellulosic biomass and consequently on fluorescence properties.

When comparing these results to those obtained for the morphology of cells, it seems that loss of fluorescence and deformation of cell walls could be related. In regard to this hypothesis, Pearson correlation coefficients were calculated between fluorescence intensities and the significant morphological parameters for beechwood samples. It showed that fluorescence intensity strongly and positively correlates with lumen circularity (r = 0.85), as well as with lumen area for which there is a high correlation for beechwood vessels (r = 0.9). Quantifying fluorescence intensity and morphological data in a larger panel than that used in the present study by varying pretreatment conditions would allow confirming the relation between chemical (as fluorescence) and morphological changes of wood.

## Conclusions

We have developed a complete and automated image processing method for rapid and easy quantification of morphological parameters and fluorescence intensity. Regarding its application, accurate quantification can only be obtained when imaging tissues can be easily sectioned and are not too damaged since deformation of cell walls could also result from the sectioning.

Investigation of two different wood species pretreated with steam explosion provided promising results. First, regarding the evolution of morphological parameters with the severity of pretreatment: while perimeter showed no significant changes, area and circularity were significantly reduced when applying the most severe pretreatments. Consequently, these two parameters can be considered as complementary markers of alteration of cells, with the area as a marker of cell size reduction and circularity as a marker of cell wall alteration. Relating the results to pretreatment severity, there was no direct correlation with the temperature of pretreatment, however an effect of the severity was visible alongside with an effect of the wood species, as spruce was deformed at a lower temperature than for beechwood. Concerning fluorescence of wood cell walls, the intensity decreased with higher severity of pretreatment. Moreover, fluorescence intensity was correlated with temperature, as well as with morphological parameters. Overall, these results highlight in detail the impact of steam explosion pretreatment at the cellular scale, in complement to other physico-chemical approaches, so that recalcitrance of biomass benefits from a relevant method to assess biomass reactivity.

In this study, the quantitative features were obtained from images acquired by fluorescence macroscopy. With minor adjustments, the method could easily be extended to other techniques such as X-ray tomography and scanning electron microscopy for quantification of morphological parameters and confocal microscopy for quantification of fluorescence. It could also be used at higher magnification to monitor fine alteration of cell walls structure and thickness. In the same way, it is not specific to steam explosion pretreatment, other types of pretreatments could be studied as well as biological degradation of wood or native model plant cell walls.

## Supplementary Information

**Additional file 1. **Mean values and standard deviations of perimeter, area and circularity of cell lumens for each sample.

## Data Availability

Data are available from the authors upon reasonable requests. The scripts for quantification of morphological parameters and fluorescence intensity are freely available on the Internet through the GitLab platform at https://gitlab.com/farelab/teamgp/publications/audibert_et_al_2023.
