# Supplementary material for: Automated quantification of fluorescence and morphological changes in pretreated wood cells by fluorescence macroscopy
Source: Plant Methods. 2023 Feb 15;19:16. doi: 10.1186/s13007-023-00991-6 (PMC9933311; doi:10.1186/s13007-023-00991-6)
Supplement: Supplementary file 1 — Additional file 1. Mean values and standard deviations of perimeter, area and circularity of cell lumens for each sample. [file 13007_2023_991_MOESM1_ESM.docx]

Additional file 1 – Mean values and standard deviations of perimeter, area and circularity of cell lumens for each sample.

| Sample | Section | Perimeter (µm) | Area (µm²) | Circularity |
| --- | --- | --- | --- | --- |
| Spruce – untreated | 1 | 73 ± 26 | 386 ± 246 | 0.79 ± 0.10 |
|  | 2 | 77 ± 28 | 399 ± 228 | 0.75 ± 0.12 |
|  | 3 | 74 ± 31 | 408 ± 275 | 0.79 ± 0.11 |
|  | 4 | 76 ± 28 | 402 ± 241 | 0.78 ± 0.10 |
|  | 5 | 81 ± 29 | 481 ± 291 | 0.81 ± 0.09 |
| Spruce – 170°C 15min | 1 | 65 ± 21 | 260 ± 145 | 0.72 ± 0.12 |
|  | 2 | 69 ± 25 | 326 ± 211 | 0.76 ± 0.11 |
|  | 3 | 71 ± 27 | 339 ± 213 | 0.76 ± 0.10 |
|  | 4 | 68 ± 22 | 309 ± 174 | 0.77 ± 0.11 |
|  | 5 | 81 ± 28 | 459 ± 274 | 0.76 ± 0.11 |
| Spruce – 190°C 15min | 1 | 59 ± 22 | 209 ± 130 | 0.69 ± 0.13 |
|  | 2 | 72 ± 33 | 286 ± 206 | 0.64 ± 0.14 |
|  | 3 | 67 ± 25 | 276 ± 185 | 0.69 ± 0.12 |
|  | 4 | 64 ± 26 | 240 ± 167 | 0.65 ± 0.12 |
|  | 5 | 88 ± 33 | 332 ± 222 | 0.61 ± 0.14 |
| Spruce – 210°C 15min | 1 | 73 ± 34 | 280 ± 201 | 0.62 ± 0.16 |

| Sample | Section | Perimeter (µm) | Area (µm²) | Circularity |
| --- | --- | --- | --- | --- |
| Beechwood – untreated | 1 | 149 ± 49 | 1324 ± 750 | 0.70 ± 0.14 |
|  | 2 | 149 ± 56 | 1368 ± 865 | 0.71 ± 0.13 |
|  | 3 | 154 ± 61 | 1402 ± 851 | 0.70 ± 0.15 |
|  | 4 | 131 ± 51 | 1133 ± 720 | 0.75 ± 0.09 |
| Beechwood – 170°C 15min | 1 | 151 ± 45 | 1507 ± 776 | 0.76 ± 0.10 |
|  | 2 | 149 ± 42 | 1387 ± 698 | 0.73 ± 0.10 |
|  | 3 | 147 ± 60 | 1537 ± 1117 | 0.76 ± 0.10 |
|  | 4 | 138 ± 48 | 1187 ± 762 | 0.71 ± 0.13 |
|  | 5 | 137 ± 47 | 1240 ± 792 | 0.75 ± 0.09 |
| Beechwood – 190°C 15min | 1 | 145 ± 42 | 1288 ± 726 | 0.72 ± 0.13 |
|  | 2 | 145 ± 52 | 1304 ± 809 | 0.72 ± 0.14 |
|  | 3 | 136 ± 39 | 1077 ± 555 | 0.68 ± 0.08 |
|  | 4 | 142 ± 42 | 1329 ± 740 | 0.76 ± 0.08 |
|  | 5 | 148 ± 47 | 1483 ± 814 | 0.78 ± 0.06 |
| Beechwood – 210°C 15min | 1 | 117 ± 27 | 555 ± 257 | 0.49 ± 0.10 |
|  | 2 | 151 ± 59 | 1020 ± 697 | 0.52 ± 0.14 |
|  | 3 | 120 ± 33 | 493 ± 256 | 0.43 ± 0.13 |
|  | 4 | 126 ± 41 | 774 ± 434 | 0.59 ± 0.13 |
|  | 5 | 155 ± 53 | 1038 ± 560 | 0.53 ± 0.13 |
